# Impact of Age on Hospital Outcomes Following Minimally Invasive Posterior Lumbar Interbody Fusion: Retrospective Analysis of the Nationwide Inpatient Sample Database from 2016 to 2020

**DOI:** 10.2196/76424

**Published:** 2026-01-06

**Authors:** Yu-Jun Lin, Fu-Yuan Shih, Jin-Fu Huang, Chun-Wei Ting, Yu-Chin Tsai, Lin Chang, Yu-Hua Huang, Ming-Jung Chuang

**Affiliations:** 1 Department of Neurosurgery Kaohsiung Chang Gung Memorial Hospital and Chang Gung University College of Medicine Kaohsiung City, Taiwan Taiwan; 2 Department of Neurosurgery Kaohsiung Municipal Ta-Tung Hospital Kaohsiung City Taiwan

**Keywords:** age, minimally invasive posterior lumbar interbody fusion, MIS-PLIF, Nationwide Inpatient Sample, NIS, in-hospital outcome

## Abstract

**Background:**

Minimally invasive posterior lumbar interbody fusion (MIS-PLIF) is commonly performed to treat degenerative lumbar spinal conditions. Patients of advanced age often present with multiple comorbidities and reduced physiological reserves, influencing surgical risks and recovery. The growing aging population has led to a rising demand for care for older adults, posing significant challenges for health care systems worldwide.

**Objective:**

This study aimed to identify the associations between different age groups and MIS-PLIF outcomes.

**Methods:**

This study retrospectively analyzed data from the United States Nationwide Inpatient Sample collected between 2016 and 2020. Patients aged ≥60 years who underwent MIS-PLIF were eligible for inclusion in this study. Patients were categorized into age groups (60-69, 70-79, and ≥80 y). Logistic and linear regressions were used to determine the associations between the study variables and outcomes, including in-hospital mortality, complications, nonroutine discharge, and length of stay.

**Results:**

A total of 785 patients aged ≥60 (mean age 69.4, SD 0.2) years who underwent MIS-PLIF were included in the analysis, and 18.7% (147/785) experienced at least one complication. After adjustment, compared with patients aged 60 to 69 years, the risk of nonroutine discharge was significantly increased in patients aged 70 to 79 years (adjusted odds ratio 2.33, 95% CI 1.57-3.46; *P*<.001) and ≥80 years (adjusted odds ratio 4.79, 95% CI 2.64-8.67; *P*<.001). No significant differences in the risk of complications or length of hospital stay were observed across the age groups.

**Conclusions:**

In older patients undergoing MIS-PLIF, advanced age is an independent predictor of nonroutine discharge. Furthermore, our findings suggest that age alone is not an independent risk factor for complications or extended hospital stays among older patients. These findings underscore that MIS-PLIF is a viable option for older patients, for whom extra attention may still be needed for postoperative care. Implementing age-stratified management for older patients undergoing MIS-PLIF may have important clinical policy implications.

## Introduction

Minimally invasive posterior lumbar interbody fusion (MIS-PLIF) represents a surgical cornerstone in addressing lumbar spine pathology [1,2]. This innovative procedure accesses the lumbar spine through a posterior approach to treat damaged intervertebral discs and fuse adjacent vertebrae [3]. The procedure can achieve results similar to those of open procedures while minimizing disruption to the surrounding tissues.

With the increase in population age, the incidence of degenerative spine disorders has also increased, leading to an increased demand for surgical treatments. Conditions such as osteoarthritis, spinal stenosis, and degenerative disc disease are more common in older individuals, resulting in a growing need for procedures such as spinal fusion, laminectomy, and discectomy to manage pain and improve mobility [4]. The relationship between advancing age and spinal health is paramount, given the potential implications of age-related changes in bone density [5,6]. These changes may not only influence the procedural success of MIS-PLIF but also impact the overall safety and effectiveness of the procedure.

Surgery for degenerative spinal conditions presents specific challenges in the older population seeking relief from symptoms [7,8]. Beyond the structural considerations of bone density, older patients often have additional health concerns and comorbidities, adding increased risk to the surgical procedure and the subsequent recovery process [9]. Understanding the relationship between age and surgical outcomes is important for tailoring surgical approaches to ensure optimal outcomes and postoperative recovery in the older population.

Despite MIS-PLIF being a common and generally well-tolerated procedure, limited research has specifically focused on the impact of age on postoperative outcomes [10,11]. The increasing prevalence of degenerative spinal diseases in older individuals makes it important to understand how age may influence the success and safety of MIS-PLIF. Furthermore, age-stratified management is becoming increasingly important due to the rapidly growing older population worldwide. This study aimed to deepen the understanding of the effect of age on outcomes by analyzing the relationship between advancing age and postoperative outcomes of MIS-PLIF using a large, nationally representative dataset from the United States [12]. Using a large, nationally representative dataset, this study provides the statistical power necessary to detect clinically meaningful differences across age groups that smaller studies may have missed. This study may offer valuable implications for both clinical practice and policy development.

## Methods

### Ethical Considerations

This study was conducted in accordance with the principles of the Declaration of Helsinki (1975) and its amendments. The protocol for this study was submitted for review to the Institutional Review Board of Chang Gung Medical Foundation (202301168B0), which granted an exemption from formal Institutional Review Board oversight. This study complies with the terms of the Nationwide Inpatient Sample (NIS) data-use agreement. The data used in this study were obtained through the Online Health Care Cost and Utilization Project (HCUP) Central Distributor. Given the anonymized nature of the data, the requirement for informed consent was consequently waived. No compensation was provided to participants.

### Data Source

This investigation drew upon data extracted from the NIS for the period of 2016 to 2020. The NIS, an initiative of the HCUP under the aegis of the Agency for Healthcare Research and Quality [13], encompasses a 20% stratified sample of inpatient admissions across 45 states and 1051 participating hospitals. The database meticulously records a wealth of patient information at the time of discharge, including primary and secondary diagnoses and procedures, dates of admission and discharge, discharge status, demographic details, and duration of hospital stay. Moreover, the NIS is equipped with statistical weights designed to facilitate the extrapolation of these findings to national patient volumes.

### Study Design

This study was designed as a population-based, retrospective analysis. All data were sourced through a request to the Online HCUP Central Distributor, which manages the database [14] (certificate HCUP-4T28I74JW). This research strictly adhered to the data-use protocols stipulated by the NIS under the HCUP. The investigation used deidentified secondary data from the NIS database; hence, there was no direct involvement of patients or the public.

### Study Population

The NIS database was searched for patients aged ≥60 years who underwent MIS-PLIF during the study period. The exclusion criteria were (1) traumatic injury; (2) injury in traffic accidents; and (3) missing values for weight, outcomes of interest, and covariates. All diagnoses and procedures were identified by the *International Classification of Diseases, Ninth Revision and Tenth Revision, Clinical Modification* (*ICD-9-CM* and *ICD-10-CM*) codes, as detailed in Table S1 in Multimedia Appendix 1. Following previous literature, we stratified age into 3 groups (ie, 60-69 y, 70-79 y, and ≥80 y) to perform comparative analyses across these categories [15,16].

### Main Outcomes and Variables

The primary outcomes included in-hospital mortality, nonroutine discharge (ie, discharged to long-term care facilities), prolonged length of stay (LOS; defined as ≥75th percentile of LOS in the study population), and complications. Complications assessed included surgical (eg, infection, dural tear, hemorrhage, hematoma or seroma, and postoperative anemia) and medical complications (eg, pneumonia, pulmonary embolism, acute kidney injury, acute myocardial infarction, and urinary retention) based on insights from our literature review and clinical experience.

Demographic variables analyzed in this study encompassed patient age, sex, race or ethnicity (categorized as White, Black, Hispanic, and other), household income, insurance status (identified by the primary payer), and whether admission occurred over a weekend. Household income quartiles were derived from the NIS, which estimates income based on the ZIP code of each patient’s residence [17]. Comorbidities were identified using *ICD-9* and *ICD-10* codes and included diabetes mellitus, osteoporosis, obesity (defined as a BMI ≥30 kg/m²), chronic obstructive pulmonary disease, renal disease, hypertension, coronary artery disease, and heart failure. Relevant codes for each comorbidity are provided in Table S1 in Multimedia Appendix 1. Charlson Comorbidity Index was calculated from individual comorbidities to represent patients’ overall severity of comorbid conditions.

Additionally, characteristics related to the hospital, such as bed size, location and teaching status, and regional location, were also collected as part of the extensive dataset available for all patients.

### Statistical Analysis

The NIS database comprises a 20% stratified sample of US annual inpatient admissions, using weighted samples, strata, and clusters to facilitate national estimates in all analyses. Data analysis was conducted using the SURVEY procedure in SAS, which is optimized for sample survey data. Descriptive statistics for patients are presented as counts (n) and weighted percentages (%) or as means with SEs. Group comparisons for categorical variables were conducted using the Rao-Scott chi-square test, whereas weighted mean differences for continuous variables were analyzed using survey methods that account for stratification, clustering, and sampling weights, ensuring valid and robust statistical inferences within the context of complex survey designs. Logistic regression analyses were performed to assess the associations between study variables and binary outcomes, with results presented as odds ratios (ORs) and 95% CI. Linear regression was performed to assess the associations between the study variables and LOS. Multivariable regression was adjusted for variables that were significant (*P*<.05) in the univariate analysis. All statistical tests were 2-sided, with *P*<.05, deemed to indicate statistical significance. The analyses were conducted using SAS (version 9.4; SAS Institute Inc).

## Results

### Patient Selection

**Figure 1:**
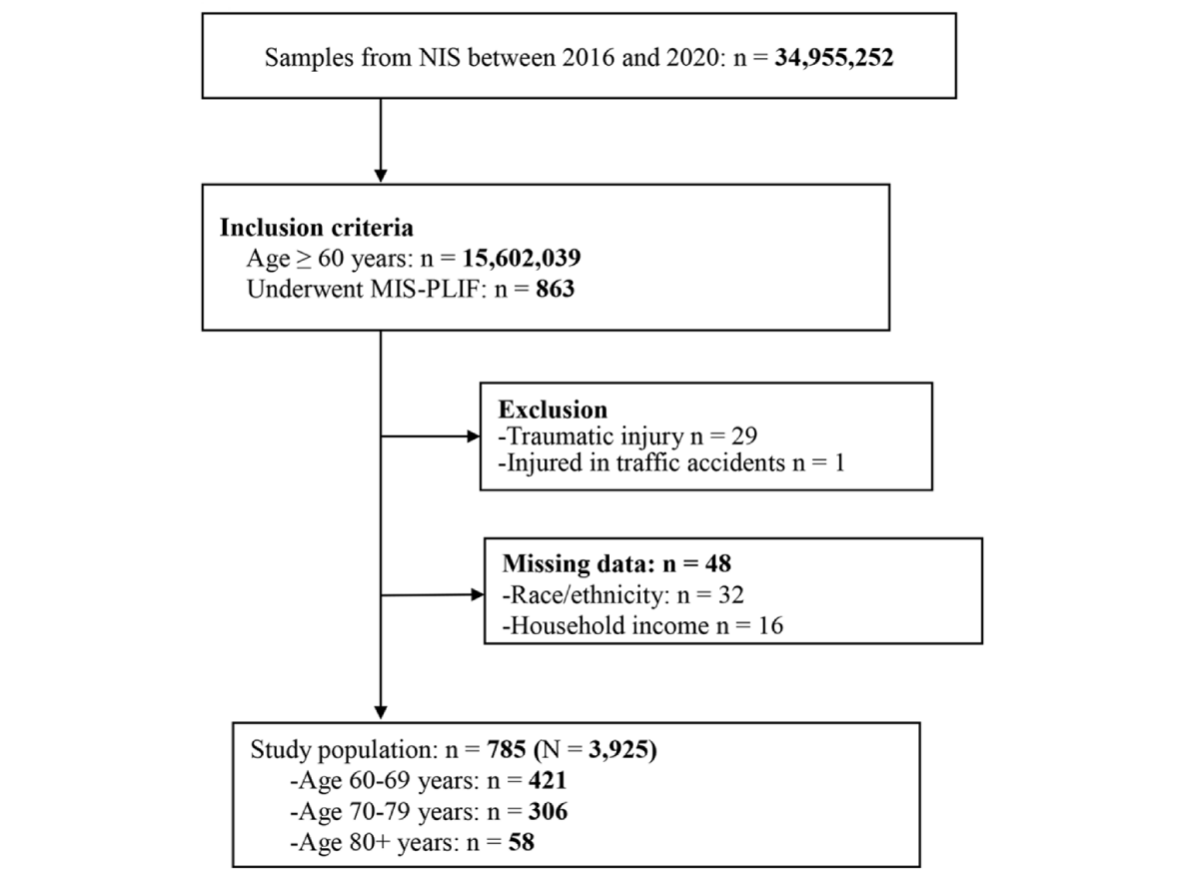
Flow diagram of patient selection. MIS-PLIF: minimally invasive posterior lumbar interbody fusion; NIS: Nationwide Inpatient Sample.

The patient selection process is illustrated in Figure 1. Between 2016 and 2020, 863 patients aged ≥60 years who underwent MIS-PLIF were identified from the NIS database. After excluding 29 patients with traumatic injuries, 1 patient with a traffic accident injury, and 48 patients with missing information on race, ethnicity, and household income, 785 patients were included in the study. Using the sample weight from the NIS database, this cohort corresponds to a total of 3925 hospitalized patients across the United States, as illustrated in Figure 1.

### Characteristics of the Study Population

In the study population, the mean age was 69.4 (SD 0.2) years, 60.9% (478/785) were female individuals, 79.2% (622/785) were White, and 72.9% (572/785) had insurance covered by Medicare or Medicaid. A total of 66% (518/785) of the patients were diagnosed with spondylolisthesis. Patients aged 60 to 69 years had a significantly higher percentage of obesity, patients aged 70 to 79 years had a significantly higher frequency of diabetes mellitus and heart failure than the other age groups, and patients aged ≥80 years had the highest frequency of renal disease and hypertension (Table 1).

**Table 1:** Characteristics of the study population by age group.

Characteristics					All patients (N=785)			Age (y)										*P* value
								60-69 (n=421)			70-79 (n=306)			≥80 (n=58)				
**Patient characteristics**																		
	Age (y), mean (SD)			69.4 (0.2)			64.5 (0.1)			73.6 (0.1)			82.7 (0.1)			*<.001^a^*		
	**Sex, n (%)**																	.95
		Male	307 (39.1)			163 (38.7)			121 (39.5)			23 (39.7)						
		Female	478 (60.9)			258 (61.3)			185 (60.5)			35 (60.3)						
	**Race and ethnicity, n (%)**																	*<.001^a^*
		Black	93 (11.8)			63 (15)			29 (9.5)			1 (1.7)						
		Hispanic	41 (5.2)			26 (6.2)			13 (4.2)			2 (3.4)						
		White	622 (79.2)			314 (74.6)			256 (83.7)			52 (89.7)						
		Other	29 (3.7)			18 (4.3)			8 (2.6)			3 (5.2)						
	**Household income, n (%)**																	*<.001^a^*
		Q1^b^	196 (25)			117 (27.8)			63 (20.6)			16 (27.6)						
		Q2	203 (25.9)			98 (23.3)			96 (31.4)			9 (15.5)						
		Q3	198 (25.2)			113 (26.8)			67 (21.9)			18 (31)						
		Q4	188 (23.9)			93 (22.1)			80 (26.1)			15 (25.9)						
	**Medicare or Medicaid insurance, n (%)**																	*<.001^a^*
		No	213 (27.1)			182 (43.2)			29 (9.5)			2 (3.4)						
		Yes	572 (72.9)			239 (56.8)			277 (90.5)			56 (96.6)						
	**Admission type, n (%)**																	.62
		Elective	713 (90.8)			383 (91)			279 (91.2)			51 (87.9)						
		Emergent	72 (9.2)			38 (9)			27 (8.8)			7 (12.1)						
	**Spondylolisthesis, n (%)**																	.71
		No	267 (34)			147 (34.9)			100 (32.7)			20 (34.5)						
		Yes	518 (66)			274 (65.1)			206 (67.3)			38 (65.5)						
**Hospital characteristics**																		
	**Hospital bed size, n (%)**																	.31
		Small	158 (20.1)			87 (20.7)			61 (19.9)			10 (17.2)						
		Medium	267 (34)			149 (35.4)			102 (33.3)			16 (27.6)						
		Large	360 (45.9)			185 (43.9)			143 (46.7)			32 (55.2)						
	**Hospital location or teaching status, n (%)**																	.11
		Rural	45 (5.7)			22 (5.2)			18 (5.9)			5 (8.6)						
		Urban nonteaching	174 (22.2)			91 (21.6)			67 (21.9)			16 (27.6)						
		Urban teaching	566 (72.1)			308 (73.2)			221 (72.2)			37 (63.8)						
	**Hospital region, n (%)**																	.27
		Northeast	155 (19.7)			79 (18.8)			63 (20.6)			13 (22.4)						
		Midwest	160 (20.4)			86 (20.4)			59 (19.3)			15 (25.9)						
		South	387 (49.3)			216 (51.3)			148 (48.4)			23 (39.7)						
		West	83 (10.6)			40 (9.5)			36 (11.8)			7 (12.1)						
	**Comorbidities, n (%)**																	
		Diabetes mellitus	199 (25.4)			100 (23.8)			91 (29.7)			8 (13.8)			*.003^a^*			
		Osteoporosis	50 (6.4)			23 (5.5)			22 (7.2)			5 (8.6)			.33			
		Obesity	198 (25.2)			116 (27.6)			72 (23.5)			10 (17.2)			*.03^a^*			
		Chronic obstructive pulmonary disease	74 (9.4)			40 (9.5)			29 (9.5)			5 (8.6)			.96			
		Renal disease	66 (8.4)			20 (4.8)			29 (9.5)			17 (29.3)			*<.001^a^*			
		Hypertension	574 (73.1)			284 (67.5)			243 (79.4)			47 (81)			*<.001^a^*			
		Coronary heart disease	116 (14.8)			54 (12.8)			50 (16.3)			12 (20.7)			.06			
		Heart failure	26 (3.3)			9 (2.1)			15 (4.9)			2 (3.4)			*<.001^a^*			
	**Charlson Comorbidity Index, n (%)**																	*<.001^a^*
		0	372 (47.4)			205 (48.7)			142 (46.4)			25 (43.1)						
		1	212 (27)			128 (30.4)			74 (24.2)			10 (17.2)						
		2	111 (14.1)			52 (12.4)			45 (14.7)			14 (24.1)						
		≥3	90 (11.5)			36 (8.6)			45 (14.7)			9 (15.5)						

^a^*P*<.05.

^b^Quartiles (Q1-Q4) were derived by dividing the distribution of the variable into four equal parts, with Q1 representing the lowest and Q4 the highest range.

### In-Hospital Outcomes

Only 0.1% (1/785) of the study population died in the hospital, whereas 18.7% (147/785) had at least one complication, of which 13.8% (108/785) had a least one surgical complication, and 7.3% (57/785) had at least one medical complication. Among the 3 age groups, patients aged ≥80 years had a significantly higher frequency of nonroutine discharge (20/58, 35% in the ≥80 y age group vs 52/421, 12.4% in the 60-69 y age group and 76/306, 24.9% in the 70-79 y age group; *P*<.001). There were no significant differences in complications (*P*=.68) and LOS (*P*=.11) between the 3 age groups (Table 2).

**Table 2:** In-hospital outcomes of the study population by age group.

Outcomes		All patients (N=785)	Age groups (y)				*P* value
			60-69 (n=421)	70-79 (n=306)	≥80 (n=58)		
All-cause in-hospital mortality, n (%)		1 (0.1)	0 (0)	1 (0.3)	0 (0)	—^a^	
Any complications, n (%)		147 (18.7)	72 (17.1)	63 (20.6)	12 (20.7)	.31	
**Any surgical complication, n (%)**		108 (13.8)	57 (13.5)	43 (14.1)	8 (13.8)	.97	
	Infection	15 (1.9)	11 (2.6)	4 (1.3)	0 (0)	—	
	Dural tear	12 (1.5)	4 (1)	6 (2)	2 (3.4)	.22	
	Hemorrhage, hematoma, and seroma	2 (0.3)	2 (0.5)	0 (0)	0 (0)	—	
	Postoperative anemia	81 (10.3)	41 (9.7)	34 (11.1)	6 (10.3)	.77	
**Any medical complication, n (%)**		57 (7.3)	25 (5.9)	27 (8.8)	5 (8.6)	.10	
	Pneumonia	3 (0.4)	1 (0.2)	1 (0.3)	1 (1.7)	.01^b^	
	Pulmonary embolism	2 (0.3)	1 (0.2)	1 (0.3)	0 (0)	—	
	Acute kidney injury	13 (1.7)	8 (1.9)	5 (1.6)	0 (0)	—	
	Acute myocardial infarction	2 (0.3)	0 (0)	1 (0.3)	1 (1.7)	—	
	Retention of urine	43 (5.5)	17 (4)	22 (7.2)	4 (6.9)	.04^b^	
Nonroutine discharge^c^, n (%)		148 (18.9)	52 (12.4)	76 (24.9)	20 (34.5)	<.001^b^	
Length of stay (d), mean (SD)		2.9 (0.1)	2.7 (0.1)	3.2 (0.2)	3.1 (0.1)	.11	

^a^Not applicable.

^b^*P*<.05.

^c^Excluding patients who died in the hospital.

### Associations Between Age and Inpatient Outcomes

Table 3 shows the univariate and multivariable analyses of the associations between patient and in-hospital outcomes. After adjustment, the multivariable analysis showed that compared with patients aged 60 to 69 years, those aged 70 to 79 years (adjusted OR 2.33, 95% CI 1.57-3.46; *P*<.001) and those aged ≥80 years (adjusted OR 4.79, 95% CI 2.64-8.67; *P*<.001) had a significantly higher risk of nonroutine discharge (Table 3).

**Table 3:** Associations between any surgical or medical complication, nonroutine discharge, and age.

Outcomes	Age (y)								
	70-79 vs 60-69					≥80 vs 60-69			
	OR^a^ (95% CI)	*P* value	Adjusted OR (95% CI)	*P* value	OR (95% CI)		*P* value	Adjusted OR (95% CI)	*P* value
Any surgical complication^b^	1.04 (0.71-1.54)	.83	1.05 (0.72-1.55)	.79	1.02 (0.55-1.90)		.95	0.98 (0.53-1.83)	.95
Any medical complication^c^	1.53 (0.99-2.38)	.06	1.16 (0.69-1.94)	.57	1.49 (0.75-2.96)		.25	0.81 (0.28-2.33)	.69
Nonroutine discharge^d,e^	2.36 (1.66-3.34)	*<.001* ^f^	2.33 (1.57-3.46)	*<.001* ^f^	3.73 (2.17-6.43)		*<.001*	4.79 (2.64-8.67)	*<.001*

^a^OR: odds ratio.

^b^Adjusted for variables that were significant (*P*<.05) in the univariate analysis, including sex, admission type, spondylolisthesis, hospital location, teaching status, and hospital region.

^c^Adjusted for variables that were significant (*P*<.05) in the univariate analysis, including sex, Medicare or Medicaid, admission type, spondylolisthesis, hospital location, teaching status, renal disease, heart failure, and Charlson Comorbidity Index (CCI).

^d^Adjusted for variables that were significant (*P*<.05) in the univariate analysis, including race, Medicare or Medicaid, admission type, spondylolisthesis, hospital location, teaching status, hospital region, diabetes mellitus, obesity, chronic obstructive pulmonary disease, renal disease, hypertension, coronary heart disease, heart failure, and CCI.

^e^Excluding patients who died in the hospital.

^f^*P*<.05.

In addition, compared to patients aged 60 to 69 years, the LOS was not significantly different in the other 2 age groups after adjustment for confounders in the multivariable analysis (70-79 vs 60-69, adjusted β=0.33, *P*=.14; ≥80 vs 60-69, adjusted β=0.14, *P*=.61). Similar findings were observed when the LOS analysis was restricted to home-discharge patients (Table S2 and S3 in Multimedia Appendix 1). Similar outcome patterns were observed when age was modeled as a continuous variable (Tables S4 and S5 in Multimedia Appendix 1).

## Discussion

### Principal Findings

This study used the NIS data to determine the impact of age on outcomes following MIS-PLIF in patients aged ≥60 years. The results revealed a low in-hospital mortality rate (only 0.1%) and a complication rate of 18.7%, with no significant differences across age groups and a similar LOS between the 3 age groups. Nonroutine discharge is a binary variable that indicates a deviation from the standard discharge pathway, such as transfer to another hospital with better medical resources, and is often associated with greater clinical severity. Notably, older age was associated with an increased risk of nonroutine discharge, with a 2.3-fold increase in patients aged 70 to 79 years and a 4.8-fold increase in those aged ≥80 years. However, older age did not significantly influence LOS or complication risk. These findings offer valuable insights into the relationship between age and postoperative outcomes after MIS-PLIF, suggesting that while MIS-PLIF is a viable and generally safe option for older patients, extra attention should be given to postoperative care, especially for those aged >70 years.

Minimally invasive procedures have become the method of choice due to decreased surgical trauma and faster recovery. Studies have compared the outcomes of MIS-PLIF with those of open procedures. For example, Mimura et al [18] compared adjacent segment pathology in patients who received a minimally invasive procedure and those who received an open approach for degenerative spondylolisthesis. The results showed that compared to conventional open PLIF, MIS-PLIF was associated with a higher disease-free survival rate and a lower incidence of adjacent segment pathology. Goldstein et al [19] performed a systematic review and meta-analysis examining adverse events of minimally invasive versus open posterior lumbar fusion. All outcomes of interest were similar between the 2 groups, although patients who underwent the minimally invasive procedures had a slightly lower rate of postoperative medical complications. However, the authors noted that the overall quality of the evidence was low.

Among the various outcomes assessed in our study, only nonroutine discharge was significantly associated with older age. Other studies have also reported that increasing age is associated with higher rates of nonroutine discharge in patients undergoing lumbar fusion; however, these studies did not further stratify older patients by age [20,21]. While there can be many reasons for this association, a possible reason is that older patients are in general more likely to be transferred to specialized care facilities after discharge because they have greater needs than their younger counterparts [22]. In addition, patients with a more advanced age may need more care than family members can provide at home or specialized rehabilitation services that are available in such facilities.

While advanced age is associated with adverse events for many surgical procedures [12,23], our results clearly showed that increased age did not significantly affect LOS or complication rate following MIS-PLIF. Luo et al [24] compared the surgical outcome of multilevel anterior cervical discectomy and fusion in older and younger patients with myelopathy. Overall, outcomes were similar between different age groups, although the older patients tended to have a lower recovery rate and higher short-term complication rate. Liu et al [25] reported that in patients undergoing PLIF, factors associated with the need for a perioperative blood transfusion were 3 or more fusion segments, low hemoglobin preoperatively, hypertension, lower BMI, and more advanced age. Our results challenge common perceptions about the risks associated with surgical procedures in older populations and highlight the potential viability of MIS-PLIF for patients of advanced age, even for those aged >80 years.

### Clinical Implications

The findings of this study have important clinical implications for the management of older patients undergoing MIS-PLIF. Notably, while MIS-PLIF is generally safe for older adults, the increased likelihood of nonroutine discharges in patients aged ≥70 years underscores the need for tailored postoperative care plans. Clinicians should consider enhanced recovery protocols and more comprehensive discharge planning to address the specific needs of older patients. This could involve early involvement of multidisciplinary teams, including geriatric specialists, physical therapists, and social workers, to facilitate smoother transitions to either home or rehabilitative settings.

### Strengths and Limitations

The primary strength of this study lies in its analysis of a large and nationally representative patient database, which enhances the generalizability of the conclusions to the broader population in the United States. This extensive scope ensures that the findings reflect a wide demographic profile, providing valuable insights applicable to the general population. The study’s relatively large sample size enabled the assessment of various complications, including infrequent complications.

Nevertheless, this study has several limitations. This was a retrospective study, which may have introduced selection bias. As with other studies relying on *ICD* coding systems, this study may have been affected by potential coding inaccuracies. Additionally, it lacks long-term postdischarge follow-up data and preadmission status information, which are essential for a comprehensive assessment of outcomes. Furthermore, comorbidities may vary in severity; however, the NIS database lacks detailed clinical parameters, such as glomerular filtration rate, glycated hemoglobin levels, and precise BMI values, limiting our ability to assess severity or perform precise staging for conditions such as diabetes, chronic kidney disease, and obesity. Complications were identified throughout the hospitalization period, without the ability to distinguish between intraoperative and postoperative events. Preoperative physical performance status, such as the American Society of Anesthesiologists Physical Status Classification, was not available. We could not confirm the timing of postoperative infections due to the absence of time-stamped data. Certain outcomes, such as changes in neurological function, altered mental status, or pain, are not captured in this administrative database, as it lacks such granular clinical and laboratory details. Minor complications, such as intolerance to pain medication, are also difficult to accurately identify, as they may not be consistently coded in electronic medical records. Although this study provides a broad overview of the outcomes using a large cohort, these limitations warrant cautious interpretation of the findings and highlight the need for future clinical studies to address more detailed outcome measures.

### Conclusions

In conclusion, this study on the impact of age on MIS-PLIF outcomes demonstrated that the procedure is relatively safe for patients aged ≥60 years, as evidenced by the low in-hospital mortality rate and low complication rate, regardless of age. However, patients aged ≥70 years had a higher risk of nonroutine discharge. Although age alone did not significantly affect LOS and complications, targeted postoperative care for older patients, particularly those aged ≥70 years, is still recommended.

These findings support the notion that MIS-PLIF is a viable surgical option for older adults, including those aged ≥80 years. Importantly, age alone should be viewed as a factor that necessitates tailored postoperative strategies. Implementing age-stratified management pathways—including early engagement of geriatric care teams, customized rehabilitation plans, and structured discharge protocols—may optimize recovery, reduce health care burden, and enhance patient outcomes.

## Appendix

Multimedia Appendix 1 Supplementary Tables S1 to S5.

## Abbreviations

HCUP: Health Care Cost and Utilization Project
ICD-9-CM: International Classification of Diseases, Ninth Revision
ICD-10-CM: International Classification of Diseases, Tenth Revision, Clinical Modification
LOS: length of stay
MIS-PLIF: minimally invasive posterior lumbar interbody fusion
NIS: Nationwide Inpatient Sample
OR: odds ratio

## Data Availability

All data generated or analyzed during this study are included in this published article and its supplementary information files.
