# Supplementary material for: Impact of Age on Hospital Outcomes Following Minimally Invasive Posterior Lumbar Interbody Fusion: Retrospective Analysis of the Nationwide Inpatient Sample Database from 2016 to 2020
Source: JMIR Med Inform. 2026 Jan 6;14:e76424. doi: 10.2196/76424 (PMC12820539; doi:10.2196/76424)
Supplement: Multimedia Appendix 1 [file medinform_v14i1e76424_app1.docx]

**Supplementary Table S1: ICD codes used in the study**

|  | ICD 10 Code |
| --- | --- |
| MIS-PLIF | **PCS**: 0SG0371, 0SG037J, 0SG03AJ, 0SG03J1, 0SG03JJ, 0SG03K1, 0SG03KJ, 0SG0471, 0SG047J, 0SG04AJ, 0SG04J1, 0SG04JJ, 0SG04K1, 0SG04KJ |
| Traumatic injury | **CM**: S00-S39 |
| Injured in traffic accidents | **CM**: V40-V49, V89 |
| Infection | **CM**: L00-L08, A00-B99, T81.43, O86.03, Z16 |
| Dural tear | **CM**: G96.11, G97.41 |
| Hemorrhage, hematoma and seroma | **CM**: M96.8, G97.3, G97.5-G97.6, R58 |
| Postoperative anemia | **CM**: D62 |
| Pneumonia | **CM**: A48.1, J12-J18 |
| Pulmonary embolism | **CM**: I26 |
| Acute renal failure | **CM**: N17 |
| Acute myocardial infarction | **CM**: I21 |
| Retention of urine | **CM**: R33 |
| Spondylolisthesis | **CM**: M43.1 |
| Diabetes mellitus | **CM**: E10-E14 |
| Osteoporosis | **CM**: M81.0, M81.8 |
| Obesity | **CM**: E66 |
| COPD | **CM**: J44 |
| Renal disease | **CM**: I12.0, I13.1, N03.2-N03.7, N05.2-N05.7, N18, N19, N25.0, Z49.0-Z49.2, Z94.0, Z99.2 |
| Hypertension | **CM**: I10-I16, I1A |
| Coronary heart disease | **CM**: I25 |
| Heart failure | **CM**: I50 |

**Supplementary Table S2: Length of stay in home discharge patients by age group**

|  | All patients  (N = 785) | **Age, years** | | |  |
| --- | --- | --- | --- | --- | --- |
| Outcomes |  | 60-69 | 70-79 | ≥ 80  (n = 58) | p-value |
|  |  | (n = 421) | (n = 306) |  |  |
| **LOS, day ^a^** | 2.36 ± 0.09 | 2.40 ± 0.11 | 2.31 ± 0.12 | 2.39 ± 0.30 | 0.840 |
| Valid n | 636 | 369 | 229 | 38 |  |

Abbreviations: LOS, length of stay

^a^ Including only home discharge patients.

**Supplementary Table S3: Association between age group and LOS in home discharge patients**

| Outcome | Age, years (vs. 60-69) | | | | | | | | |
| --- | --- | --- | --- | --- | --- | --- | --- | --- | --- |
|  | 70-79 | | | |  | >= 80 | | | |
|  | Beta (95% CI) | p-value | aBeta (95% CI) | p-value |  | Beta (95% CI) | p-value | aBeta (95% CI) | p-value |
| **LOS ^a, b^** | -0.85 (-0.38, 0.21) | 0.565 | -0.08 (-0.41, 0.26) | 0.652 |  | -0.00 (-0.61, 0.61) | 0.997 | 0.08 (-0.53, 0.69) | 0.795 |

Abbreviations: CCI, Charlson Comorbidity Index; LOS, length of stay; aBeta, adjusted Beta; CI, confidence interval.

p-values < 0.05 are shown in bold.

^a^ Adjusted for variables that were significant (p < 0.05) in the univariate analysis, including Medicare/Medicaid, admission type, spondylolisthesis, hospital location/ teaching status, and CCI.

^b^ Including only home discharge patients.

**Supplementary Table S4: Associations between continuous age and any surgical complication, medical complication, and non-routine discharge**

| **Outcomes** | **Main Predictor: Age (cont.)** | | | |
| --- | --- | --- | --- | --- |
|  | **OR (95% CI)** | **p-value** | **aOR (95% CI)** | **p-value** |
| **Any surgical complication ^a^** | 0.99 (0.97, 1.03) | 0.911 | 1.00 (0.97, 1.02) | 0.879 |
| **Any medical complication ^b^** | 1.04 (0.99, 1.08) | 0.071 | 0.95 (0.88, 1.03) | 0.213 |
| **Non-routine discharge ^c, d^** | 1.08 (1.05, 1.11) | **<0.001** | 1.10 (1.07, 1.14) | **<0.001** |

Abbreviations: CCI, Charlson Comorbidity Index; OR, odds ratio; aOR, adjusted odds ratio; CI, confidence interval; NA, no event occurred in one group.

p-values < 0.05 are shown in bold.

^a^ Adjusted for variables that were significant (p < 0.05) in the univariate analysis, including sex, admission type, spondylolisthesis, hospital location/ teaching status, and hospital region.

^b^ Adjusted for variables that were significant (p < 0.05) in the univariate analysis, including sex, Medicare/Medicaid, admission type, spondylolisthesis, hospital location/teaching status, renal disease, heart failure, and CCI.

^c^ Adjusted for variables that were significant (p < 0.05) in the univariate analysis, including race, Medicare/Medicaid, admission type, spondylolisthesis, hospital location/ teaching status, hospital region, diabetes mellitus, obesity, chronic obstructive pulmonary disease, renal disease, hypertension, coronary heart disease, heart failure, and CCI.

^d^ Excluding patients who died in the hospital.

**Supplementary Table S5: Associations between continuous age and LOS**

| **Outcomes** | **Main Predictor: Age (cont.)** | | | |
| --- | --- | --- | --- | --- |
|  | **Beta (95% CI)** | **p-value** | **aBeta (95% CI)** | **p-value** |
| **LOS ^a, b^** | 0.03 (-0.00, 0.06) | 0.053 | 0.01 (-0.01, 0.04) | 0.298 |

Abbreviations: CCI, Charlson Comorbidity Index; LOS, length of stay; aBeta, adjusted Beta; CI, confidence interval.

p-values < 0.05 are shown in bold.

^a^ Adjusted for variables that were significant (p < 0.05) in the univariate analysis, including Medicare/Medicaid, admission type, spondylolisthesis, hospital location/ teaching status, and CCI.

^b^ Excluding patients who died in the hospital.
